# Shared decision-making in patients with multiple sclerosis

**DOI:** 10.3389/fneur.2022.1063904

**Published:** 2022-11-11

**Authors:** Dirk T. Ubbink, Olga C. Damman, Brigit A. de Jong

**Affiliations:** ^1^Department of Surgery, Amsterdam University Medical Centers, Public Health Research Institute, University of Amsterdam, Amsterdam, Netherlands; ^2^Department of Public and Occupational Health, Amsterdam University Medical Centers, Public Health Research Institute, Free University of Amsterdam, Amsterdam, Netherlands; ^3^Department of Neurology, Amsterdam University Medical Centers, MS Center Amsterdam, Amsterdam Neuroscience Research Institute, Public Health Research Institute, Free University of Amsterdam, Amsterdam, Netherlands

**Keywords:** multiple sclerosis, shared decision-making, value-based healthcare, patient-centered care, patient empowerment

## Abstract

Multiple sclerosis (MS) is a chronic and progressive neurological disorder impacting physical, cognitive, and psychosocial health. The disease course, severity, and presence of symptoms differ within and between persons over time and are unpredictable. Given the preference-sensitive nature of many key decisions to be made, and the increasing numbers of disease-modifying therapies, shared decision-making (SDM) with patients seems to be key in offering optimum care and outcomes for people suffering from MS. In this paper, we describe our perspective on how to achieve SDM in patients with MS, following key SDM-elements from established SDM-frameworks. As for deliberation in the clinical encounter, SDM communication training of professionals and feedback on their current performance are key aspects, as well as encouraging patients to participate. Concerning information for patients, it is important to provide balanced, evidence-based information about the benefits and the harms of different treatment options, including the option of surveillance only. At the same time, attention is needed for the optimal dosage of that information, given the symptoms of cognitive dysfunction and fatigue among MS-patients, and the uncertainties they have to cope with. Finally, for broader communication, a system is required that assures patient preferences are actually implemented by multidisciplinary MS-teams. As SDM is also being implemented in many countries within the context of value-based health care, we consider the systematic use of outcome information, such as patient-reported outcome measures (PROMs) and Patient Decision Aids, as an opportunity to achieve SDM.

## Introduction

Multiple sclerosis (MS) is the most common cause of non-traumatic neurologic disability in young adults in the Western world. Worldwide, an estimated 2.8 million people suffer from this disease, while in Western Europe roughly 200 per 100,000 inhabitants live with MS (www.atlasofms.org). The pathological hallmarks are neuroinflammation, demyelination, and neurodegeneration (1). It is a progressive, incurable disorder of the central nervous system (CNS) leading to a broad range of symptoms, i.e., motor, sensory, visual, balancing, cognitive, and urogenital problems (1). MS typically presents itself with episodes of disability (i.e., relapses) followed by phases of recovery, which is called relapsing-remitting MS (RRMS). In general, RRMS evolves into secondary progressive MS (SPMS), which is characterized by steady disease progression over time. Approximately 15% of patients present a (slowly) progressive disease course from the onset: primary progressive MS (PPMS) (1). Besides physical limitations, invisible symptoms are often present, including cognitive dysfunction and fatigue, that have a profound impact on quality of life (QoL) and major consequences for personal, social, and occupational functioning (2, 3). The disease course, severity, and presence of symptoms differ within and between persons over time and are largely unpredictable. MS comes with substantial medical costs and loss of productivity for patients and society. Medical costs include hospitalization, patient care, and medication. Loss of productivity costs is related to episodes of disability, long-term disability, absences from work, and unemployment. Due to these facts, it is the second most expensive chronic disorder in the United States (4).

Over the past three decades, disease-modifying treatments (DMTs) have become increasingly available to reduce irreversible CNS damage, clinically aiming to decrease the frequency of relapses and slow down disability progression (5). Today, DMTs are classified as either moderate efficacy or high efficacy DMTs, and they all differ in route, timing, and location (i.e., hospital vs. home) of administration, safety profile, and tolerability. Data have shown that early and more aggressive treatment may prevent future disability (6). However, these therapies do not cure the disease; the efficacy varies between different subtypes of MS and may change over the years, and may be accompanied by side effects (5, 7, 8). On an individual level, there is uncertainty about the efficacy and safety of DMTs, and this needs to be monitored intensively. Aiming to combine high-efficacy treatment with a good safety profile, many patients switch several times from one DMT to another during the course of their disease (9). Not surprisingly, adherence to DMTs is problematic for a large number of patients, especially in the longer term (10, 11).

Because of the erratic course of the disease, the availability of a vast number of pharmacological and non-pharmacological treatment options, the frequent introduction of new therapeutic options, and the uncertainty of the effectiveness of drug therapies, the treatment decisions are preference-sensitive in nature (12). Therefore, it is of the utmost importance to involve patients in the treatment decisions to be made. Although many neurologists and other professionals involved in MS care may recognize this need, there is yet no structural approach toward engaging patients in the decision-making process, based on their individual values and outcomes. However, a modern concept in medicine, called shared decision-making (SDM), can be helpful to support this development and alleviate the situation of these patients. This paper presents an evidence-based viewpoint as to the care for patients with MS using this concept.

In order to deliver high-quality, patient-centered care, SDM is seen as the preeminent model to make clinical decisions together with patients. It is a collaborative way of decision-making, whereby health professionals and patients mutually exchange information and thoughts to arrive at a treatment decision that best fits the patient (13). Not only is SDM driven by the ethical imperative of patient autonomy, the benefits of SDM go beyond patient-centeredness: scientific studies show that it may result in better health outcomes, better adherence to DMTs, and higher patient satisfaction (12, 14, 15). In this paper, we describe our perspective on how to achieve SDM in patients with MS, following established SDM frameworks.

## General SDM elements

**Figure 1:**
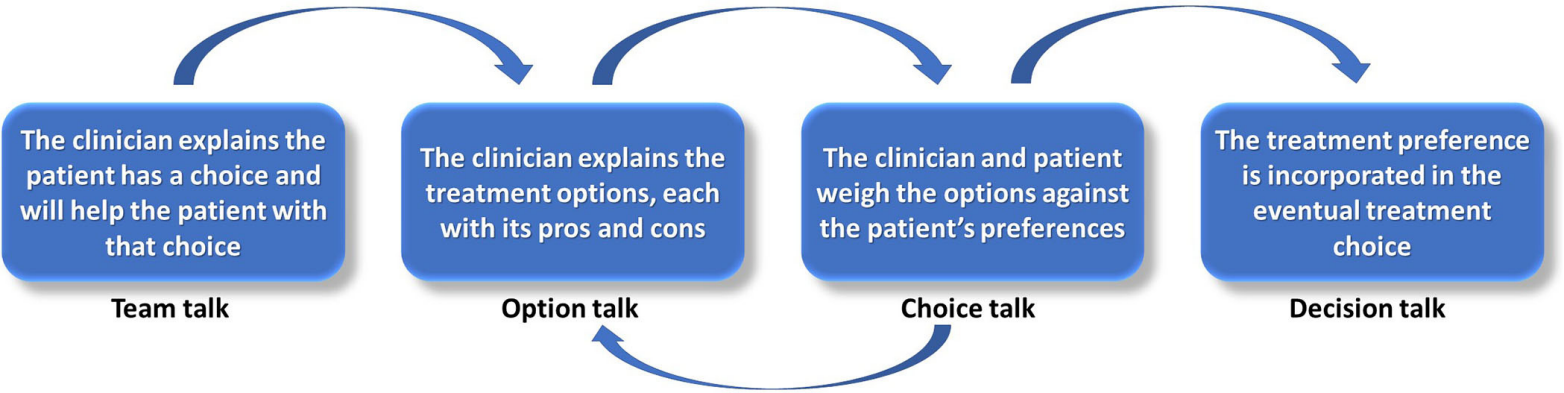
The four steps of shared decision-making in clinical encounters.

The general principles of SDM have been outlined in the literature (16, 17). We discern three key elements (17). The first element is *deliberation in the clinical encounter* between patients and professionals about the options available. This can be structured along the following steps (Figure 1): Inform the patient there is a choice to be made and the patient's vote is essential in this, and the clinician will help the patient understand the choices (‘Team talk'); explain the options with the pros and cons of each option ('Option talk'); explicitly invite the patient to share his/her preferences and values regarding the options to arrive at a preference (‘Choice talk'), and; finally incorporate the patient's preference in the eventual treatment decision (‘Decision talk').

**Figure 2:**
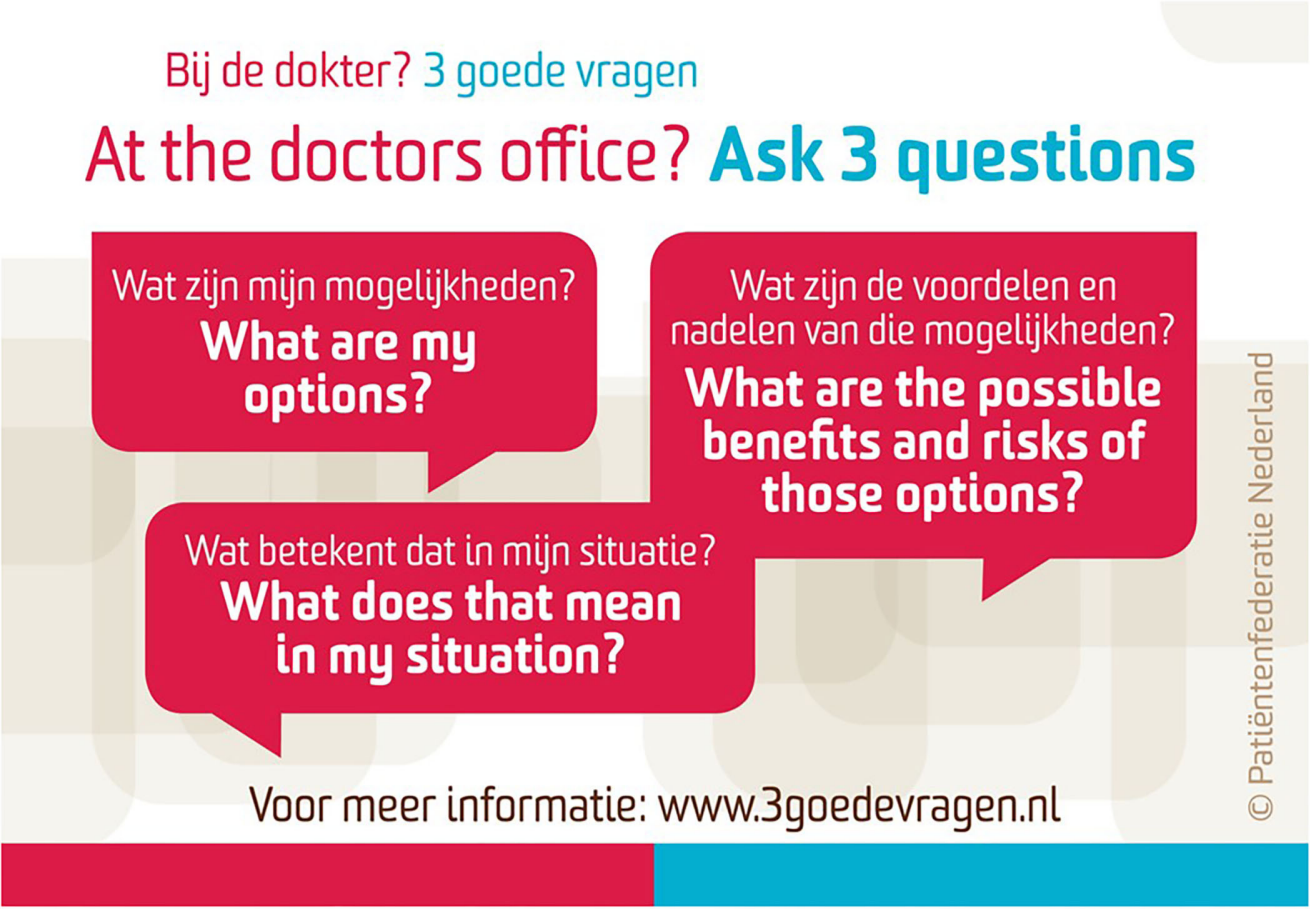
The ‘three good questions' card.

Professionals are usually well-trained in informing the patient about their disease and the preferred treatment options for this, based on the healthcare provider's point of view. The other SDM-specific parts should be taught through SDM-communication skills training (18). In our institution, this training takes half a day and allows healthcare professionals to practice their communication skills with a simulation patient (professional actor) along the steps of SDM, guided by a medical psychologist, and based on a clinical case the participants may submit themselves. Also, feedback sessions based on audio-recordings of decision-making consultations can improve SDM (19). Patients can be encouraged to participate in the SDM process by trained professionals, but also by supplying them with supporting materials, e.g., the three-good-questions cards (Figure 2) based on the AskShareKnow communication model (20), with the aim to be better prepared for the clinical encounter.

A second key element to facilitate SDM, inside and outside the clinical encounter, is to disclose high-quality *information for patients*, for example using decision-making support tools based on the International Patient Decision Aids Standards (21). Patient Decision Aids (PtDAs; delivered online, as video, or on paper) for people who are facing treatment decisions result in better communication between patients and healthcare providers. These tools may also improve satisfaction related to the decision (22). For patients, PtDAs provide reliable, balanced, and evidence-based information about the options and the benefits and harms of those options, including uncertainties (23, 24). PtDAs also typically provide so-called value clarification methods (VCMs) that help patients think about the desirability of options or attributes of options within a specific decision context, to identify the preferred option (25, 26). PtDAs may be rather elaborate and, although they are helpful to prepare for the clinical encounter at home, they do not necessarily induce SDM between professionals and patients. For the clinical encounter itself, so-called option grids have been developed to present the available evidence on frequently asked questions during the consultation (27). Recently, the use of individual outcome data, such as based on patient-reported outcome measures (PROMs), has been suggested to provide information relevant to SDM, since these data also stipulate issues that matter to patients (22, 28–30). For the provision of all these information types and tools, an urgent question has become how to make them useful for patients with lower health literacy and numeracy (31–33). To really ensure that the most essential information for the decision can be used by patients with lower health literacy, more seems to be needed than modifications to written and verbal information (33).

A final key element is the *broader communication system*, to ensure that patients' preferences are actually implemented. Often, organizational changes are required to facilitate SDM, such as allocating time to spend on SDM-conversations in busy outpatient clinic schedules, and ICT-support to facilitate the collection of PROMs and distribution of PtDAs. Addressing broader organizational and system-level factors in which key communications between professionals as well as between professionals and patients are embedded, is thought to be essential to ensure that SDM becomes a normalized part of healthcare practice (30, 34, 35).

## What is already known about SDM in patients with MS?

The idea of SDM is increasingly embraced in clinical guidelines for the treatment of MS internationally, although not all guidelines explicitly address this as SDM (36–38). Just like for many other diseases, studies have shown that the vast majority of patients with MS prefer the SDM model over other models, such as the paternalistic model (39, 40). In addition, treatment preferences and goals have been shown to differ between patients and clinicians, which is a reason per se to apply SDM (12, 41, 42). Patients' goals tended to focus on the impact of specific symptoms on their day-to-day lives, whereas providers' goals focused on slowing down the course of disease progression (43). A recent Finnish study found that patients with MS desire to be better informed and more involved in the decision-making process. Unfortunately, however, they seldom experienced the information provided by clinicians to be helpful and, hence, search through other digital information sources (44). A British qualitative study among patients with MS suggested that patients may benefit from PtDAs that structure first and consecutive treatment decisions (45, 46). On the other hand, obtaining this overview of treatment options can be confronting to patients, especially when overwhelmed by uncertainty about the effects of DMT and fear about transitioning to secondary progressive MS (46).

As described by Colligan et al. (12) many countries employ initiatives to develop PtDAs to facilitate SDM with patients with MS; most of which focus on disease modification drugs. A Dutch study investigated the cost-effectiveness of SDM in patients with MS, including the use of a PtDA about DMTs (45). They found a potential cost-effectiveness of SDM for DMTs, especially if SDM would lead to the continuation of treatment. PtDAs have been developed for patients with common MS-related impairments (i.e., fatigue, cognitive dysfunction, reduced mobility, work- and study-related issues) to help them compare their options. Examples can be found on the MStrust website. However, these PtDAs are hardly applied during regular medical encounters. So far, it is unclear whether these might influence the outcomes in general, including patient participation and an overall sense of autonomy and empowerment.

## Key elements for SDM with patients with MS

Based on what is known from the general SDM field as well as from studies focusing on MS, we advocate the key SDM elements as described above are addressed in the following manner.

### Deliberation in the clinical encounter

SDM communication training of professionals and feedback on their current performance are key aspects to ensure deliberation in the clinical encounter, as well as encouraging patients to participate. There is increasing attention for SDM training in MS care (47). In general, we presume health professionals in this field are fairly skilled in the phase of ‘choice talk', which includes value clarification. Since MS usually starts between the ages of 20 and 40, many people combine their illness with family and working life, have questions about whether to get pregnant, what job or education to follow, can I still bring my children to school, or contribute optimally to work meetings, etc. Such priorities and issues in patients' lives are likely a standard element that are discussed in clinical encounters. However, the next step, i.e., weighing the options with the patient to arrive at a shared decision, may be more difficult to clinicians. Although professionals may know quite well-how to discuss such patient values important for deliberation, there are often no tools to prepare patients. Employing PROMs in the care path may facilitate this (48). In addition, PtDAs may better inform patients when the ‘option talk' is not conducted optimally.

### Information for patients

Key decisions where information is needed for patients with MS and their healthcare providers are:

- starting, stopping, or switching DMTs
- starting, stopping, or switching symptomatic non-pharmacological treatments, including physical therapy, cognitive rehabilitation therapy.
- starting, stopping of symptomatic pharmacological therapy, including fampridine (a drug that may improve mobility)

For each of these decisions it is important to provide balanced, evidence-based information about the benefits (i.e., improvements in symptoms or disease progression), the harms (i.e., risk of side-effects and longer-term problems), and the route of administration of different treatment options, including the option of surveillance only. For the use of DMTs specifically, often a decision has to be made, first about whether to start medication, and later on about what type of medication. As a result, structuring the decision and giving overviews (‘decision maps') can be of importance. Especially the information about DMT options is complex, as they have different disease-modulating profiles, variations in efficacy, safety, dosage frequency, and route of intake. It seems a complex task for patients to absorb this information and to integrate this with individual values/priorities, such as how to deal with comorbidity, or is the use of medication compatible with a child-wish, etc. In addition, the knowledge about the effects of switching DMTs is still developing (49), and this particular decision can be difficult to make (50).

An essential question is to what extent all benefits and harms, including working mechanisms and the more seldom side-effects, need to be explained in a PtDA and/or option grid, considering what is known about the difficulties in using such information among patients with lower health literacy or numeracy (51, 52). The benefit of DMT options can be difficult to convey clearly, for example: “In a group of 100 patients taking a DMT, a 30% reduction of relapses will occur as compared to taking no medication.”

Based on previous research (46, 50), the following attributes should be taken into account:

- effect on number of relapses each year (benefit of treatment)
- effect on severity and recovery of relapses (benefit of treatment)
- effect on disease progression (benefit of treatment)
- how easy it is to take the treatment (e.g., injections versus pills, where administrated, frequency of blood tests needed for monitoring, etc.)
- side-effects with high probability of occurrence, but may be temporary (e.g., lymphopenia, flushing)
- severe side-effects with low probability of occurrence (e.g., progressive multifocal leukoencephalopathy)
- long-term effects on QoL (e.g., cognitive functioning, societal and work participation)
- how treatment fits in with pregnancy wish

Information providers should consider the question regarding how much quantitative information should be provided to patients who are more vulnerable in information processing, such as those with lower health literacy or numeracy. Specifically for patients with MS, another influencing factor is their possible cognitive dysfunction and fatigue, which may further complicate engagement with the information about benefits and harms.

Besides evidence-based risk communication, VCMs are important when providing information. There is no best practice as to the design of VCMs (21). In designing such VCMs for patients with MS, clinicians may ask patients to evaluate the attributes as described above, in terms of “How important is the effect of the medication on reducing relapses/ease of use/the risk of a complication for you?,” using rating or ranking scales. An alternative is to ask patients to answer open questions like “What are important things for you in daily life that may affect your decision?.” Identifying the outcome(s) that an individual patient considers important may be helpful to prioritize such aspects for patients with MS, since many patient-experienced symptoms will influence patients' preferences toward or away from certain treatment options.

### Broader communication system

It is essential to develop a system that ensures the implementation of patients' preferences by multidisciplinary MS-teams. Because of the wide range of complaints and symptoms, many specialties in and outside the hospital are involved in the care of MS patients. Close collaboration between those specialties and using the same, up-to-date evidence is desirable to implement patients' preferences.

## Key preconditions for implementing SDM for patients with MS

To align healthcare decisions with a patient's unique situation and preferences both patients and healthcare professionals should be well-informed. This concerns being knowledgeable about the general clinical characteristics (i.e., disease and symptoms), and the outcomes of possible treatment options, but also explicitly about the specific context, values, and preferences of the individual with MS.

Training of professionals in SDM, particularly in the ‘option' and ‘choice' talks.

Well-designed PtDAs and/or option grids need to be available, which structure a large amount of information for patients (e.g., using decision maps) and with risk communication that is accessible to people from various intellectual backgrounds. Knowledge is needed about what the right timing is to provide such tools to patients with MS and how to tailor the information to the specific decision situation of the patient.

If PROMs are used, they need to be integrated into electronic medical records and sent out timely, before the clinical encounter. PROMs-questionnaires need to be suitable for various levels of health literacy. Ideally, this should result in on-screen availability of the individual results during the medical encounter (53, 54), and is presented as a dashboard with well-designed timelines, tables, and graphs in the EMR.

## Discussion

### Future perspectives

In summary, based on the available literature on MS and other diseases combined with local experiences, we expect that the introduction of the principles of SDM in the MS-clinic will lead to higher QoL, improve clinical outcomes, increase patient engagement, and lower the use of resources as compared to usual care. The actual benefits of a value-based care model in MS as to clinical and patient-reported outcomes, patient engagement, and resource usage are still to be assessed. Future studies are also needed to elucidate which outcome information really works to promote SDM among patients with MS. Our and other experiences and results may hopefully be beneficial to improve current guidelines and to other MS-centers across the world.

## Data availability statement

The original contributions presented in the study are included in the article/supplementary material, further inquiries can be directed to the corresponding author/s.

## Author contributions

DU, OD, and BJ contributed to conception and design of the study. DU wrote the first draft of the manuscript. OD and BJ wrote sections of the manuscript. All authors contributed to manuscript revision, read, and approved the submitted version.

## Conflict of interest

The authors declare that the research was conducted in the absence of any commercial or financial relationships that could be construed as a potential conflict of interest.

## Publisher's note

All claims expressed in this article are solely those of the authors and do not necessarily represent those of their affiliated organizations, or those of the publisher, the editors and the reviewers. Any product that may be evaluated in this article, or claim that may be made by its manufacturer, is not guaranteed or endorsed by the publisher.
